# Tribological and Rheological Properties of Poly(vinyl alcohol)-Gellan Gum Composite Hydrogels

**DOI:** 10.3390/polym14183830

**Published:** 2022-09-14

**Authors:** Yang Feng, Shao-Cong Dai, Khoon Lim, Yogambha Ramaswamy, Ahmad Jabbarzadeh

**Affiliations:** 1 School of Aerospace, Mechanical, and Mechatronic Engineering, University of Sydney, Camperdown, NSW 2006, Australia; 2 Department of Orthopaedic Surgery and Musculoskeletal Medicine, University of Otago, Christchurch 8140, New Zealand; 3 School of Biomedical Engineering, University of Sydney, Sydney, NSW 2006, Australia

**Keywords:** hydrogel, gellan gum, polyvinyl alcohol, PVA composites, tribology, rheology, tissue engineering, cartilage tissue repair

## Abstract

Polymeric poly(vinyl alcohol) (PVA)-based composite hydrogels are promising materials with various biomedical applications. However, their mechanical and tribological properties should be tailored for such applications. In this study, we report the fabrication of PVA-gellan gum (GG) composite hydrogels and determine the effect of GG content on their rheological and tribological properties. The rheology tests revealed an enhanced storage (elastic) modulus with increased gellan gum (GG) concentration. The results showed up to 89% enhancement of the elastic modulus of PVA by adding 0.5 wt% gellan gum. This elastic modulus (12.1 ± 0.8 kPa) was very close to that of chondrocyte and its surrounding pericellular matrix (12 ± 1 kPa), rendering them ideal for cartilage regeneration applications. Furthermore, the friction coefficient was reduced by up to 80% by adding GG to PVA, demonstrating the increased elastic modulus improved chance of survival under mechanical shear stresses. Examining PVA/GG at different concentrations of 0.1, 0.3, and 0.5 wt% of GG, we demonstrate that at a load of 5 N, the friction coefficient decreases by increasing the GG concentration. However, at higher loads of 10 and 15 N, a 0.3 wt% concentration was sufficient to significantly reduce the friction coefficient. For PVA and PVA/GG composites, we observed a reduction in friction coefficient by increasing the load from 5 to 15 N. We also found the friction to be independent of the sliding velocity. Possible mechanisms of achieving a reduced friction coefficient are discussed.

## 1. Introduction

Hydrogels are hydrophilic cross-linked polymeric networks, and can absorb a significant amount of aqueous medium [1]. Hydrogels are one of the promising soft materials in tissue engineering, due to their excellent biocompatibility and structural similarity to the biological extracellular matrix (ECM) [2,3]. They have been extensively explored for soft tissue applications, including cartilage tissue engineering. The articular cartilage is a biphasic material and consists of collagen and glycosaminoglycan (GAG), a high molecular weight polymer that swells in an environment with an excessive aqueous phase [4]. The pressure in a fully operational joint is ~0.1–10 MPa, and the elastic moduli of chondrocyte cells and the surrounding matrix responsible for cartilage regeneration are reported to be 9–12 kPa; designing materials with similar moduli will enhance their regeneration capabilities [5]. Therefore, hydrogels are considered to be promising soft materials for replacing or regenerating injured cartilage due to their biocompatibility and ability to regenerate new tissue [4,5,6,7,8,9]. Hydrogels can be designed to meet tissue engineering, mechanical, and tribological requirements. For tissue engineering applications, the elastic modulus of hydrogel should promote cellular adhesion, proliferation, and biomimetic microenvironment, and be close to the modulus of cells it is designed to promote [10,11]. In addition, the hydrogels should also possess a low friction coefficient and good mechanical properties that allow load-bearing under operational conditions in joints. Therefore, we assume some extra support will be needed to lower the load in such a setting. The primary motivation for analyzing the tribological and rheological properties of the hydrogels in this study is to develop a biosynthetic hydrogel composite suitable for tissue regeneration, with low friction to reduce the likelihood of hydrogel degradation due to shear forces.

While polymer gels are widely used as lubricants [12] in industrial applications, a hydrogel is not a lubricant but provides good lubricity due to its soft nature and ability to hold significant water content. This renders it ideal for use in biomedical applications. The tribological behaviour of hydrogels is hypothesized to be very similar to that of the articular cartilage, which provides low friction lubrication by combining biphasic and boundary lubrications. The biphasic lubrication theories include boosted lubrication [13], fluid load support [14], and a more recent tribological rehydration model [15]. Boundary and interfacial molecular lubrication models such as hydration lubrication [16,17] concentrate on the role of water and hydrated polymeric components at the atomic level. According to the fluid support model, the fluid trapped in the porous-elastic structure of materials such as cartilage and hydrogels is pressurized under load and prevents further deformation. Solid and fluid phases share the load, and friction is low as long as the fluid supports part of the load. Once this fluid load support vanishes, the friction will increase. Experiments have revealed that friction increases with time for sliding under continuous loading conditions [14,18]. This is attributed to the loss of fluid and its support for loading. Recently, photoelastic experiments have questioned the role of the fluid load support model in the low friction lubrication of hydrogels [19]. They have shown that under continuous loading and sliding, the load support by the fluid and solid phases does not change while the friction increases. They have argued that other friction mechanisms should be considered to explain the low friction in hydrogel systems.

Various composite hydrogels with improved biocompatibility and mechanical properties have been explored for tissue engineering applications. In recent years, it has been shown that blending synthetic polymers with natural materials can improve hydrogels’ mechanical properties and increase biological activity [2,20,21,22,23,24,25,26,27,28]. Poly(vinyl alcohol) (PVA)is a synthetic hydrophilic (-OH group) polymer and has been considered a promising material for various biomedical applications. However, their lack of cell adhesion moieties limits their application. These limitations are overcome by incorporating natural polymers or proteins to enhance their biocompatibility [15], making them suitable for tissue engineering applications [2,20,21,22,23,24,25,26,27,28]. Gellan gum (GG) is an anionic extracellular polysaccharide that consists of tetrasaccharide repeat units containing β-D-glucose, β-D-glucuronic acid, and α-L-rhamnose monomers in a 2:1:1 molar ratio. The GG hydrogel remains stable during long-term culture in standard media, and does not suffer unwanted dissolution due to ionic exchange [4,25]. GG is considered a suitable material for cartilage tissue engineering applications due to its structural similarity with some of the GAG components present in the cartilage tissue matrix.

PVA chains have several hydroxyl pendant groups that have been utilized to functionalize them with various functional groups, including methacrylates. The functionalization of the pendant groups with methacrylates will enable photopolymerization and rapid hydrogel formation in the presence of a photoinitiator [25]. A similar functionalization of natural polymers such as GG, as well as combining them with functionalized PVA and photocrosslinking them, will result in the formation of biosynthetic hydrogels with enhanced mechanical property, structural integrity and biocompatibility [26].

Previous studies showed that blending PVA hydrogel with GG and Ca^2+^ ions improved some of its mechanical properties and improved bioactivity [2]. However, the rheological and tribological properties of PVA/GG composite hydrogels are not well-explored. This study investigates the tribological and rheological properties of poly(vinyl alcohol) (PVA)/GG. We aim to understand the effects of adding various concentrations of GG to form PVA/GG hydrogels, and to investigate their rheological and tribological properties under conditions relevant to physiological sliding speeds.

## 2. Materials and Methods

### 2.1. Synthesis of PVA Methacrylates and GG Methacrylates

The PVA used to fabricate the hydrogels was 98% hydrolyzed PVA (Sigma-Aldrich, St. Louis, MO, USA, Mw~13,000–23,000). In preparing the 10 wt% PVA solution, 50 g PVA powder was added into 500 mL distilled water while heating at 80 °C and mixed until fully dissolved, and methacrylic anhydrate was added. The pH value of the solution was adjusted to 8 using 1 M NaOH and 1 M HCl and then left to react for 18 h. The solution was purified again by ultrafiltration through a 10 kDa molecular weight cutoff membrane for five days. The solution was then lyophilized to obtain the dry product.

GG was functionalized with methacrylates as previously described [27]. In this process, GG (Gelrite, Sigma, Formula Weight: 1000 kg/mol) was dissolved in deionized water at 90 °C for 1 h, methacrylate anhydride was added, and the reaction was continued for 6 h with the constant maintenance of the pH at 8.0. Finally, the solution was purified by dialysis and lyophilized for fabricating the hydrogels.

### 2.2. Fabrication of Composite PVA/GG Hydrogels

In this work, the hydrogels were formulated with 15% (*w*/*w*) total macromer concentration in water. PVA-MA and GG-MA were dissolved separately by heating them at 80°C. The PVA/GG co-hydrogels were formed by mixing PVA-MA with GG-MA and photopolymerizing the blend with a UV light source at an intensity of 30 mW/cm^2^ in the presence of photoinitiator, Irgacure 2959, which was added to create a final concentration of 0.1% (*w*/*w*). PVA-MA hydrogels were used as controls, and PVA/GG hydrogels with three different concentrations of 0.1, 0.3, and 0.5% (*w*/*w*) of GG were used for analysis.

### 2.3. Rheological Tests

Rheological properties were measured by an Anton Paar MCR-302 rheometer (Anton Paar GmbH, Graz, Austria) using anti-slip parallel plate geometry with a 25 mm plate. Hydrogel samples of 25 mm diameter and 1 mm thickness were prepared in all cases. Initially, amplitude sweep tests for strains (γ) between 0.1–100% were conducted at a frequency of *f* = 1 Hz (angular frequency *ω* = 2π*f* = 6.28 rad/s), storage modulus G′ was measured, and the limit of the linear viscoelastic regime (LVR) was determined. It is essential to conduct the frequency sweeps within the LVR. Subsequently, frequency sweeps at a strain of γ = 0.1% were conducted for 0.1–100 rad/s angular frequency. The gap size in all frequency sweep tests was 1 mm. The registered normal force in all cases was positive. Finally, we conducted all tests at 37 °C for pure PVA and PVA/GG composites. The temperature was controlled through a Peltier system integrated with the rheometer. Strong signals with torques of 25–40 μNm well above the machine’s minimum torque sensitivity (0.5 nNm) were registered in all cases, ensuring the reliability of the G′ values. In addition, the G″/G′ ratio was well above the recommended value of 0.01, ensuring G″ values were reliable.

### 2.4. Tribology Tests

**Figure 1:**
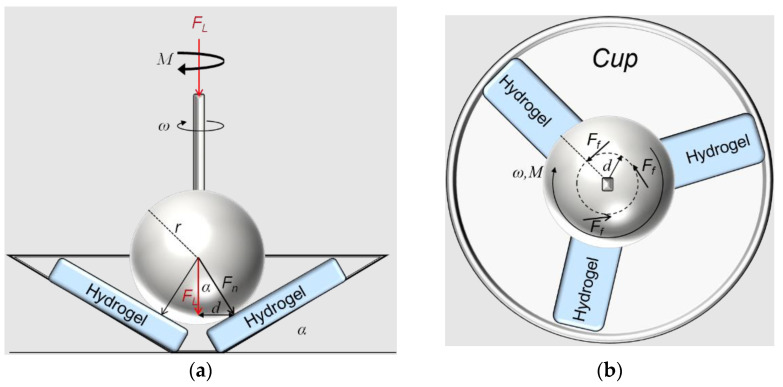
Schematic (**a**) side and (**b**) top views of the ball-on-three-plates tribology test.

In the tribological tests, the friction coefficient of PVA/GG hydrogels was measured by conducting a ball-on-three-plates test using an Anton Paar^®^ MCR-302 machine mounted with a T-PTD 200 tribology measuring cell. The tribology cell was designed to measure the friction in dry and lubricated contacts with different materials and lubricants [29,30]. Temperature control was achieved using a Peltier system that kept the temperature constant at 37 °C. The measuring cell included a shaft with a ball mounted on one end. The ball formed three-point contacts with the three hydrogel samples fitted radially at equal intervals in a holder cup. The samples had an inclined orientation (see Figure 1). The cup could be filled with lubricant to perform lubricated tribology tests. The MCR-302 machine controlled the load and rotating velocity of the shaft. The machine also measured the friction force. The counter surface (mounted ball) could be changed. Here we used a steel ball with the grade of 100 Cr6 for all measurements. The average roughness of the ball surface was 0.1 µm. Figure 1 shows the working mechanism of the ball-on-three-plates test by using the T-PTD 200 tribology measuring cell.

The shaft applied a load of *F*_L_ on the probing ball that made contact with three hydrogel samples. Three hydrogel samples of 15.5 × 6 × 3 mm dimensions were mounted on inclined surfaces and radially positioned at equal 120° angular intervals in a circular cup. The shaft applied a load that transferred normal force F_N_ on each of the three samples. The machine applied a load *F_L_* to the shaft, and the normal load on each sample could be established using the incline angle α. Therefore, a normal load of *F_N_ = F_L_/*3*cosα* was applied to each hydrogel sample. A probing steel ball of radius *r* = 6.35 mm made contact with the hydrogel samples at a radial distance *d = rsinα*. The ball was mounted on a shaft whose rotational velocity, *ω*, was controlled by the machine. Therefore, the linear velocity of the ball could be calculated as v_s_ = *rd* = *rωsinα*, where *ω* is the rotational velocity in rad/s. The sliding distance was calculated from the rotational velocity, time, and distance *d*. The average friction force can be calculated from the measured torque *M*, as *F_f_* = *M*/3*d*. Therefore, the friction coefficient μ can be calculated as μ = *F_f_*/*F_N_*, and the reported friction coefficient is the average friction coefficient of the three samples used in each test.

The load and sliding speed were chosen based on the data available for various human activities to relate the experimental results to the applications. The loads chosen in this study were *F_L_* = 5, 10, and 15 N, corresponding to *F_N_* = 2.36, 4.71, and 7.07 N loads on each sample. Studying hydrogels for various applications requires load consideration. For example, in the hydrogels studied for opthalmological applications, the contact pressures during tribological tests were nearly an order of magnitude larger than the intended pressure between the eyelid and cornea [31]. For the loads examined here, the calculated pressures based on the Hertzian contact model are ~5–12 kPa. These are lower than the pressures expected in a joint (0.3–10 MPa), which has a modulus of approximately 0.5 MPa. Cartilage carries loads 20 times higher than its compressional modulus [15]. Large pressures will be possible by formulating hydrogels with a higher modulus. However, hydrogels with such a high modulus will not be suitable for tissue regeneration. Therefore, we assume that these hydrogels will be used in a setting where extra support will reduce the joint’s pressure. Part of the load will be supported by fluid pressure, similar to the working mechanism of cartilage [15]. The sliding speeds experienced in the human knee during activities, such as walking and running, are calculated using Rennie’s model [31]. The speed values varied from 0.05 m/s to 0.4 m/s [31,32]. Therefore, the velocities chosen for the tribological test were 0.05, 0.1, 0.2, and 0.4 m/s, which are within this range. Tribological tests for pure PVA and PVA/GG composites were conducted at three different normal loads of *F_N_* = 5, 10, and 15 N. In all experiments, the hydrogel samples were submerged in distilled water. The running time for each experiment was 10 min. Each experiment was conducted twice, and the average friction coefficient of the three samples which were used in each test was measured and recorded. The average results of the two tests on each experiment are presented here. Therefore, the friction coefficients reported here are the averages over six samples of the same material.

## 3. Results and Discussions

### 3.1. Rheological Properties

**Figure 2:**
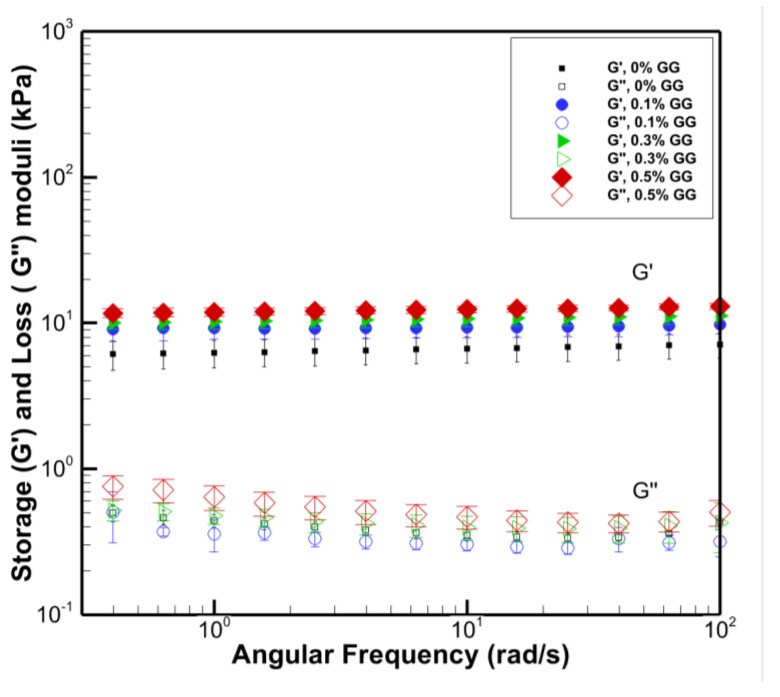
Storage modulus G′ and loss modulus G″ for pure PVA and PVA/GG hydrogels with different GG contents.

Figure 2 shows the storage (elastic) modulus G′ and loss modulus G′′ versus the angular frequency. The results show the average of several measurements in the linear viscoelastic regime. The average G′ values for pure PVA, 0.1%, 0.3%, and 5% PVA/GG composites are 6.4 ± 1.3; 9.3 ± 1.5; 10.0 ± 1.1; and 12.1 ± 0.8 kPa, respectively. This shows a consistent increase of 45%, 62%, and 89% in average G′ when the concentration of GG is increased. Although the average G′ of pure PVA is 45% lower than the PVA/GG hydrogel with 0.1% GG, the statistical uncertainties are large, and we cannot conclude a meaningful difference. However, for PVA/GG with 0.3% GG, there is at least an increase of 37% in the G′ values considering statistical uncertainties. However, the increase of the GG content to 0.5% results in a considerable increase in G′. The average G′ values show an increase of 89% for the 0.5% PVA/GG samples. Even considering the statistical uncertainty in the results, there is at least a 48% increase in G′. The storage modulus G′ is a measure of the stored energy in the deformed material and relates to the elastic part of the viscoelastic behaviour. The higher G′ of the hydrogel means it can store more energy during the deformation, and that the material’s stiffness is higher [33,34]. Therefore, the results suggest that increasing the GG content to a certain level enhances the stiffness of the PVA/GG hydrogels.

The improved modulus values are within 10–30 kPa, suggested for promoting osteogenic cell differentiation [11]. Furthermore, the elastic modulus of single chondrocytes cells is reported to be 9.3 ± 0.8 kPa [5]. The elastic modulus of chondrons (chondrocyte and its surrounding pericellular matrix) is reported to be 12 ± 1 kPa [5]. Therefore, the composite PVA/GG gels’ elastic moduli are very close to the chondrogenic matrix, making them ideal for cartilage regeneration and other similar tissue engineering applications.

Figure 2 also shows the loss modulus G″ for PVA and PVA/GG hydrogels with different GG contents. The loss modulus is a measure of the viscosity of the viscoelastic material. The results show that the G′ > G″ and both are independent of the frequency. The average values of G″ are 0.43 ± 0.05;0.40 ± 0.08; 0.47 ± 0.09, and 0.61 ± 0.12 kPa for 0%, 0.1%, 0.3%, and 0.5% GG content, respectively. There is no statistically significant effect on loss modulus by adding 1% and 3% GG to PVA. However, G″ shows some marginal increase for the GG content to 0.5 wt%. The loss modulus G″ describes the viscous part of the viscoelastic behaviors, which can be defined as the liquid-state behaviour of the sample [34]. Wang and his coworkers studied the mechanical properties of PVA/GG composites for higher concentrations of GG from 0.5 to 10 wt%. They have shown that the water content of PVA/GG hydrogels increases with an increase in the mass fraction of GG [2]. They attributed this behaviour to GG’s ability to prevent the mutual entanglement of PVA polymer chains and the destruction of the original microcrystalline zones of PVA [35]. They also identified that increasing GG content resulted in a higher capacity for water retention [36]. These properties of the GG in PVA/GG hydrogels would allow more water to be absorbed and retained in the hydrogel, and higher water content can result in a higher loss modulus G′′ of the hydrogel.

**Figure 3:**
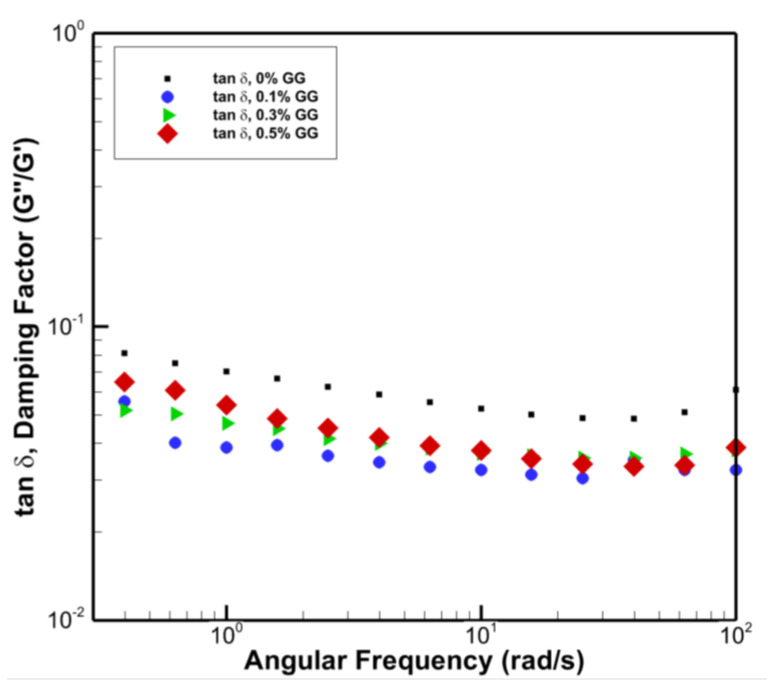
Damping factor (tanδ) versus angular frequency for pure PVA and PVA/GG composites with different concentrations.

The damping factor (tanδ) is the tangent of the phase angle (δ) between the strain wave and stress wave and is measured as the ratio of G″/G′. For a completely viscous liquid, tanδ is close to 1 (δ = 90°), and for true elastic material, tanδ is close to 0 (δ = 0°). We have plotted tanδ versus angular frequency in Figure 3. The average values for the damping factor (tanδ) for the pure PVA and its composites of 0%, 0.1%, 0.3%, and 0.5% GG content are 0.068 (δ = 3.9°), 0.043 (δ = 2.5°), 0.046 (δ = 2.6°), and 0.051(δ = 1.2°), respectively. In all gels, we observe the dominance of elastic behaviour. The lower values of δ for PVA/GG gels also show improved elasticity with adding GG to the PVA.

### 3.2. Tribological Properties

The analysis of the tribology tests is divided into three sections, which include an investigation of the effects of GG content, the load, and the sliding velocity on the friction of PVA/GG hydrogels.

#### 3.2.1. Effect of Gellan Gum Content on the Friction of PVA/GG Hydrogels

**Figure 4:**
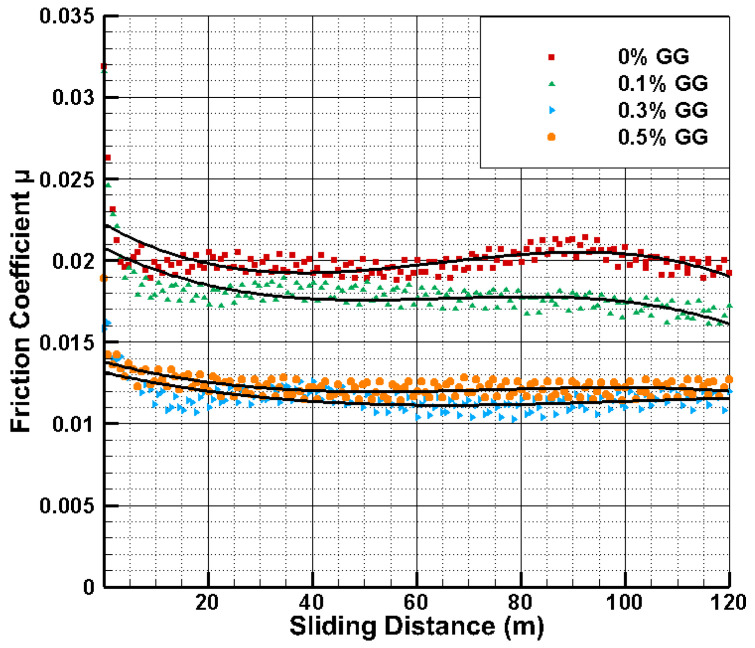
Friction coefficient versus sliding distance for PVA/GG hydrogels of different GG content under the load of 10 N and the sliding velocity of 0.2 m/s. The solid lines are guides to the eye.

The measurements for each test were done over 600 s, and data were collected at every sec. The data obtained from the tribometer include the sliding distance and the friction coefficient at each time step. The friction coefficient is plotted against the sliding distance (Figure 4) for pure PVA and PVA/GG hydrogels of three different GG contents under the load of 10 N and the sliding velocity of 0.2 m/s. For all cases, the friction coefficient remains relatively stable over 10 min of sliding. For flat-on-flat tribological contacts with a stationary contact area, or in the absence of lubricating fluid, the hydrogels and soft tissues (e.g., cartilage) exhibit increased friction over sliding time. This is due to the loss of fluid in the sample and the lack of fluid support. However, recent work by Moore et al. [15] has demonstrated that forming a convergent wedge using a curved contact results in “tribological rehydration” of cartilage, whereby the friction remains low and stable. Moore et al. [15] have coined the cSCA term for convergent Stationary Contact Area for such a configuration. The tribological contact in our test is similar to a convergent stationary contact area. Therefore, a ball on the three-plate geometry used here produces a stable friction coefficient for hydrogels during sliding. We believe this is due to the same convergent wedge-induced “tribological rehydration,” which pushes the fluid back into the porous media and retains continuous low friction sliding. Conducting the tests in water also provides the opportunity for quick rehydration and prevents drying that may lead to increased friction. This eliminates the effect of dehydration of hydrogel on friction, and the focus can be shifted to other properties.

Under these conditions, the average friction coefficient of PVA hydrogel is about μ~0.021. A low friction coefficient for PVA hydrogel has previously been reported [19,20]. In particular, Porte et al. [19] have reported a μ~0.02 for PVA for migrating contact area (MCA) and tribological contact. In that tribological setting, the fluid loss in the contact is replenished as the loading is relieved, and low friction persists over long sliding distances. This situation is very similar to our case, in which a continuous supply of lubricant and cSCA geometry provides similar low friction behavior over a long sliding time.

Figure 4 shows that adding GG can significantly reduce the friction coefficient down to 80% resulting in μ~0.012. This very low friction coefficient is comparable to those of biological surfaces such as cartilages of animal joints, which have friction coefficients in the range of 0.001–0.03 [20]. Figure 4 shows that the friction coefficient decreases by increasing the GG content from 0.1 to 0.3 wt%. However, the friction coefficient reaches a limiting value of approximately ~0.012 at 0.3 wt% and does not change significantly by increasing the GG content to 0.5 wt%. A similar tendency is found in the effect of GG content under different loading conditions and the same sliding velocity.

**Figure 5:**
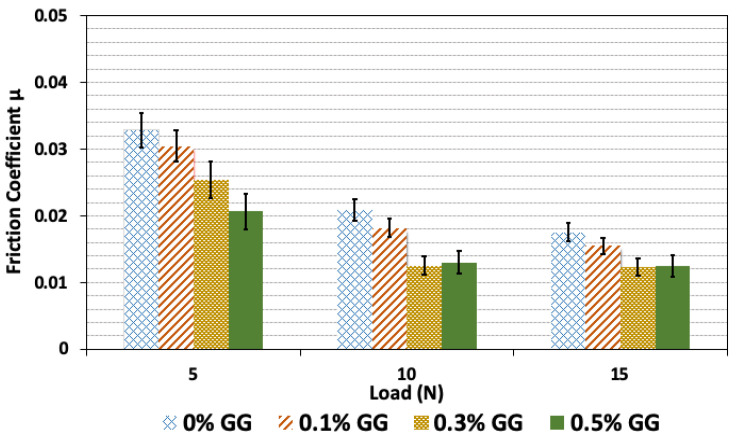
The average friction coefficient, μ, for PVA and PVA/GG hydrogel with different GG contents. The results are shown for measurements under three different loads and at a sliding velocity of 0.2 m/s.

In Figure 5, the friction coefficients are compared at a sliding velocity of 0.2 m/s for pure PVA and three different concentrations of GG in PVA/GG composite hydrogels under loads of 5, 10, and 15 N. Increasing the GG content at the lowest load of 5 N would decrease the friction coefficient. For 10 N and 15 N loads, increasing the GG content would decrease the friction coefficient; however, 0.3 wt% GG content seems to be optimally sufficient, and increasing the content to 0.5 wt% would not reduce the friction further.

#### 3.2.2. The Effect of Load on Friction

**Figure 6:**
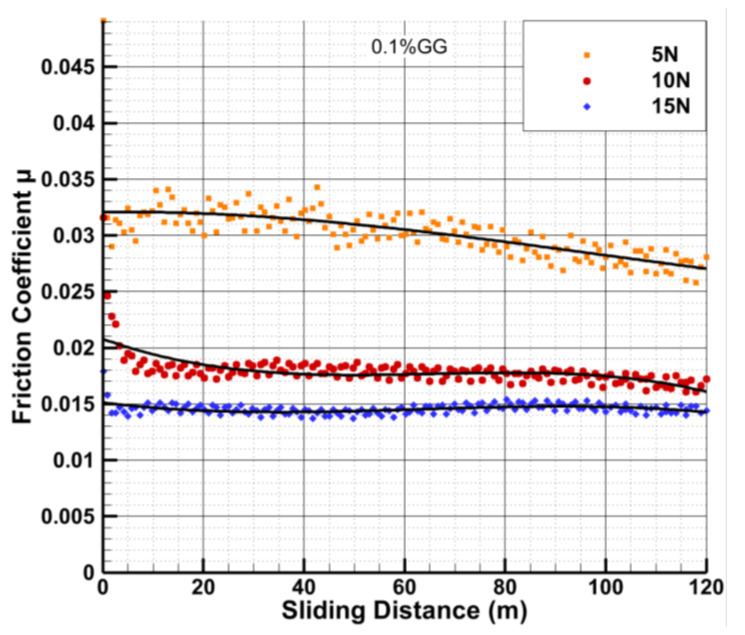
Friction coefficient versus sliding distance for the PVA/GG hydrogels with 0.1 wt% GG under the sliding velocity of 0.2 m/s. The results are shown for three different loads of 5, 10, and 15 N.

Figure 6 shows the trend in friction coefficient as a function of sliding distance during tribological testing at a sliding velocity of 0.2 m/s and under different loads of 5, 10, and 15 N for the PVA/GG hydrogels with 0.1 wt% GG content.

Figure 6 indicates that the friction coefficient for the lowest load of 5 N drifts slightly lower with time. However, at higher loads of 10 N and 15 N, the friction coefficient remains relatively stable. It also illustrates that the friction coefficient decreases with increasing the applied load, and is the highest at the lower load of 5 N. This is consistent with the dependence of the friction coefficient on the load shown in Figure 5 for PVA/GG hydrogels with various GG contents.

The hydrogel can be defined as a network of hydrophilic polymer chains that can absorb and retain a large amount of water. The PVA/GG hydrogels studied in our work contain approximately 80% water [2]. For all hydrogels tested, the friction is reduced by increasing the load. It is generally assumed that the primary reason for a hydrogel’s extremely low friction coefficient is water’s hydrodynamic lubrication and fluid load support. Therefore, hydrogels that absorb more water can provide better lubrication. When the applied load increases, there is higher pressure, which means more water would be squeezed out of the hydrogel matrix and act as the lubricant to provide better lubrication conditions between the hydrogel and its counterparts [32]. Furthermore, the hydrogels with GG content have a higher elastic modulus and will be squeezed less. This advantage is more significant at the lower load of 5 N. While this weeping lubrication explanation might sound reasonable, the tests are done in water and cannot be fully responsible for reducing the friction coefficient with the load.

Gong et al. have reported a decrease in the friction coefficient with load for various hydrogels, including PVA and gellan gum [20,21], on their own. For PVA, they have observed the same load dependence in measurement in air and water. They have demonstrated that this observation cannot be explained by hydrodynamic, weeping, or boundary lubrication models, and other surface-dependent properties should be considered. The load dependence of the friction coefficient for GG hydrogels is reported to be weaker than that of PVA [21]. Gong et al. [21] have suggested that surface-surface interaction between the gel and the counter surface may have influence here, and gels with more attractive interactions show more dependence on the load. By measuring adhesion force on the gel surface, they have shown that, while PVA has a weak attractive force, gellan gum has a weak repulsive force. This might explain the weaker dependence of friction on load (5 to 10 N) for PVA/GG composites compared to the pure PVA (see Figure 5).

#### 3.2.3. The Effect of Sliding Velocity on the Friction Coefficient

**Figure 7:**
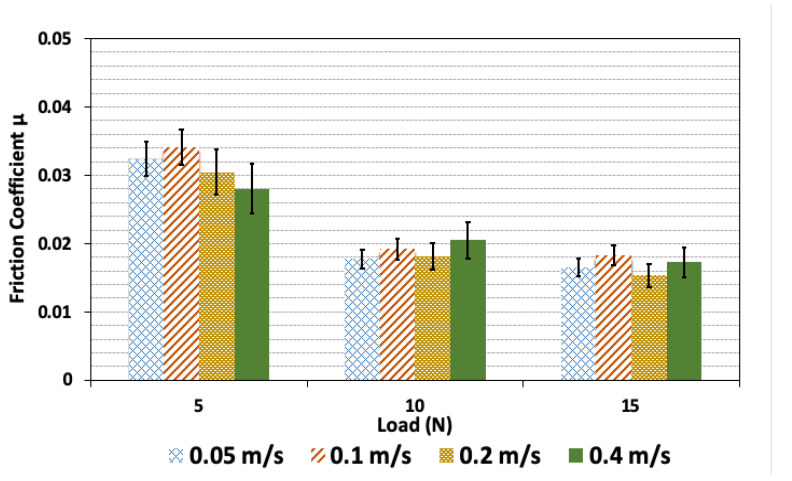
Friction coefficient of PVA/GG hydrogel with 0.1 wt% GG for different sliding velocities of 0.05, 0.1, 0.2 and 0.4 m/s. The results are shown for three different loads of 5, 10, and 15 N.

Figure 7 shows the friction coefficient of PVA/GG hydrogel with 0.1 wt% GG at different sliding velocities. The results are shown for the three loadings of 5, 10, and 15 N. While some weak dependence on the velocity at 5 N can be seen, the friction coefficient is independent of the sliding velocity in the range of those examined here for all other loading conditions. We note that for pure PVA and PVA/GG hydrogels with 0.3 and 0.5 concentrations tested, the friction coefficient for all loads is independent of the sliding velocity, and the slight differences are within the statistical uncertainty.

Reduction in friction coefficient with the sliding speed for PVA hydrogels is reported by Gong et al. [20,21] and others [37]. However, they have explained this through the adsorption-desorption model. An increase in friction with sliding velocity has been reported in the experiments conducted by Accardi et al. [18]. Porte et al. [19] suggest that this high friction results from non-replenished tribo-contact used with the tests that involved increasing the amplitude of the oscillatory sliding tests. Gong et al. [20,21] suggest that the friction force for attractive gels is due to the combination of viscous forces in hydrodynamic lubrication and elastic forces due to the deformation of adsorbed polymer chains. The elastic component is dominant at a lower velocity, and the viscous component is dominant at a higher velocity. Some weak dependence on velocity for the lowest load may indicate this behaviour, as the friction decreases with the velocity. The characteristic critical velocity v_f_ for the transition from elastic to hydrodynamic lubrication depends on the gel’s elastic modulus and temperature and v_f_~E^2/3^. However, we did not observe any dependence on sliding velocity for hydrogel with higher GG concentrations (0.3% and 0.5% GG), which had a higher modulus. Therefore, we concluded in our experiments that there was not much dependence on the sliding velocity. This could be due to the higher velocities used in our experiments.

These results indicate that increasing the GG content in PVA/GG hydrogel can reduce the friction coefficient up to a specific limit. Gong et al. [21] compared the tribological properties of pure GG hydrogel and pure PVA hydrogels. They report that GG hydrogels had a lower friction coefficient than pure PVA hydrogels. Despite this, it is surprising to see a significant improvement in the friction coefficient of pure PVA by adding only a small amount of GG.

As mentioned previously, a study by Wang and his coworkers has revealed that increasing the GG content increases the water retention capacity of PVA/GG hydrogels [2]. Besides, the results of rheological tests in this study indicate that increasing the GG content even below 0.5% would increase the storage and loss moduli (G′, G″), supporting Wang and his coworkers’ findings.

The tribological behavior of hydrogels is very similar to that of cartilage, where a combination of fluid load support and boundary lubrication mechanisms drives low friction performance. When more water is absorbed and retained in the hydrogel, it will provide better lubrication and fluid load support, leading to a lower friction coefficient. A full explanation may require further studies on how the GG changes the properties of the surface. Recently, the fluid support model for hydrogels has been questioned by Porte et al. [19]. They conducted careful photoelastic experiments on PVA hydrogels and demonstrated that the friction remains low in the absence of fluid load support under some conditions. They argued in favor of surface lubricants as one of the contributing factors in low friction behavior. Considering the significant improvement in the friction of PVA/GG hydrogel, even for very small content, we cannot rule out the effect of GG on developing more efficient surface lubricants. The mechanism for such an effect requires careful examination of the surface properties of this hydrogel and is the subject of our further investigations.

## 4. Conclusions

Preparing hydrogels that possess mechanical properties that can enhance cell growth while surviving the mechanical stresses is the key to their application in biomedical engineering. We demonstrated that adding a small percentage of gellan gum could improve elastic modulus and lower the friction of PVA/GG hydrogels. The elastic modulus of 12.1 ± 0.8 kPa for a 0.5% GG content was very close to the modulus of chondrocyte and its surrounding pericellular matrix (12 ± 1 kPa), showing good potential as a tissue engineering material for chondrocyte cell growth and cartilage repair. We also found significantly lower friction coefficients under sliding velocities relevant to those in the human knee joint. Adding gellan gum to PVA reduced the friction coefficient to as low as μ~0.012, resulting in improvement by up to 80%. This revealed that due to lower friction, these hydrogels have a good chance of surviving shear stresses in biomedical applications, providing an environment for cell growth.

Furthermore, we demonstrated that increasing the applied load would result in a decrease in the friction coefficient. The low friction behaviour was independent of the sliding velocities in the ranges relevant to daily human activities such as walking and running. The compatible modulus and low frictional properties of PVA/GG hydrogels show their potential in repairing cartilage, as well as in other biomedical applications.

## Data Availability

Not applicable.

## References

[1] Wichterle O., Lim D. (1960). Hydrophilic gels for biological use. Nature.

[2] Wang F., Wen Y., Bai T. (2016). The composite hydrogels of polyvinyl alcohol–gellan gum-Ca^2+^ with improved network structure and mechanical property. Mater. Sci. Eng. C.

[3] Li Y., Rodrigues J., Tomas H. (2012). Injectable and biodegradable hydrogels: Gelation, biodegradation and biomedical applications. Chem. Soc. Rev..

[4] Benya P.D., Shaffer J.D. (1982). Dedifferentiated chondrocytes reexpress the differentiated collagen phenotype when cultured in agarose gels. Cell.

[5] Nguyen B.V., Wang Q.G., Kuiper N.J., El Haj A.J., Thomas C.R., Zhang Z. (2010). Biomechanical properties of single chondrocytes and chondrons determined by micromanipulation and finite-element modelling. J. R. Soc. Interface.

[6] Holloway J.L., Lowman A.M., Palmese G.R. (2010). Mechanical evaluation of poly (vinyl alcohol)-based fibrous composites as biomaterials for meniscal tissue replacement. Acta Biomater..

[7] Spiller K.L., Maher S.A., Lowman A.M. (2011). Hydrogels for the repair of articular cartilage defects. Tissue Eng. Part B Rev..

[8] Yamaoka H., Asato H., Ogasawara T., Nishizawa S., Takahashi T., Nakatsuka T., Koshima I., Nakamura K., Kawaguchi H., Chung U.I. (2006). Cartilage tissue engineering using human auricular chondrocytes embedded in different hydrogel materials. J. Biomed. Mater. Res. Part A.

[9] Passaretti D., Silverman R.P., Huang W., Kirchhoff C.H., Ashiku S., Randolph M.A., Yaremchuk M.J. (2001). Cultured chondrocytes produce injectable tissue-engineered cartilage in hydrogel polymer. Tissue Eng..

[10] Wu S., Hua M., Alsaid Y., Du Y., Ma Y., Zhao Y., Lo C.Y., Wang C., Wu D., Yao B. (2021). Poly (Vinyl Alcohol) Hydrogels with Broad-Range Tunable Mechanical Properties via the Hofmeister Effect. Adv. Mater..

[11] Chaudhuri O., Gu L., Klumpers D., Darnell M., Bencherif S.A., Weaver J.C., Huebsch N., Lee H.P., Lippens E., Duda G.N. (2016). Hydrogels with tunable stress relaxation regulate stem cell fate and activity. Nat. Mater..

[12] Gorbacheva S.N., Yadykova A.Y., Ilyin S.O. (2021). Rheological and tribological properties of low-temperature greases based on cellulose acetate butyrate gel. Carbohydr. Polym..

[13] Walker P.S., Dowson D., Longfield M.D., Wright V.E.R.N.A. (1968). “Boosted lubrication” in synovial joints by fluid entrapment and enrichment. Ann. Rheum. Dis..

[14] Ateshian G.A. (2009). The role of interstitial fluid pressurization in articular cartilage lubrication. J. Biomech..

[15] Moore A.C., Burris D.L. (2017). Tribological rehydration of cartilage and its potential role in preserving joint health. Osteoarthr. Cartil..

[16] Klein J. (2013). Hydration lubrication. Friction.

[17] Jabbarzadeh A., Wandelt K. (2018). Tribological Properties of Interfacial Molecular Films. Encyclopedia of Interfacial Chemistry Surface Science and Electrochemistry.

[18] Accardi M.A., Dini D., Cann P.M. (2011). Experimental and numerical investigation of the behaviour of articular cartilage under shear loading—Interstitial fluid pressurization and lubrication mechanisms. Tribol. Int..

[19] Porte E., Cann P., Masen M. (2019). Fluid load support does not explain tribological performance of PVA hydrogels. J. Mech. Behav. Biomed. Mater..

[20] Gong J.P. (2006). Friction and lubrication of hydrogels—Its richness and complexity. Soft Matter.

[21] Gong J., Iwasaki Y., Osada Y., Kurihara K., Hamai Y. (1999). Friction of gels. 3. Friction on solid surfaces. J. Phys. Chem. B.

[22] Koyano T., Minoura N., Nagura M., Kobayashi K.I. (1998). Attachment and growth of cultured fibroblast cells on PVA/chitosan-blended hydrogels. J. Biomed. Mater. Res. Off. J. Soc. Biomater. Jpn. Soc. Biomater. Aust. Soc. Biomater..

[23] Kumar A., Han S.S. (2017). PVA-based hydrogels for tissue engineering: A review. Int. J. Polym. Mater. Polym. Biomater..

[24] Wu G., Zhao C.H., Wang C., Zhang W. (2008). The effect of preparation methods on tribological properties of PVA-H/HA composites. Iran. Polym. J..

[25] Ferris C.J., Gilmore K.J., Wallace G.G. (2013). Modified gellan gum hydrogels for tissue engineering applications. Soft Matter.

[26] Silva-Correia J., Oliveira J.M., Caridade S.G., Oliveira J.T., Sousa R.A., Mano J.F., Reis R.L. (2010). Gellan gum-based hydrogels for intervertebral disc tissue-engineering applications. J. Tissue Eng. Regen. Med..

[27] Nafea E., Poole-Warren L., Martens P. (2015). Bioactivity of permselective PVA hydrogels with mixed ECM analogues. J. Biomed. Mater. Res. Part A.

[28] Xu Z., Li Z., Jiang S., Bratlie K.M. (2018). Chemically modified gellan gum hydrogels with tunable properties for use as tissue engineering scaffolds. ACS Omega.

[29] Moreira-Izurieta F., Jabbarzadeh A. (2017). Tribological studies in cartilaginous tissue of lamb synovial joints lubricated by distilled water and interstitial-fluid-like solution. Tribol. Ind..

[30] (2014). Instruction Manual for Tribology Measuring Cell T-PTD200.

[31] Rennie A.C., Dickrell P.L., Sawyer W.G. (2005). Friction coefficient of soft contact lenses: Measurements and modeling. Tribol. Lett..

[32] Wu G., Wang C., Zhang W. (2007). The factors of speeds and loads on the tribological properties of PVA-H/HA composites. J. Appl. Polym. Sci..

[33] Meyers M.A., Chawla K.K. (2008). Mechanical Behavior of Materials.

[34] Mezger T.G. (2015). Applied Rheology: With Joe Flow on Rheology Road.

[35] Dai L., Liu X., Tong Z. (2010). Critical behavior at sol–gel transition in gellan gum aqueous solutions with KCl and CaCl_2_ of different concentrations. Carbohydr. Polym..

[36] Mahajan H.S., Gattani S.G. (2009). Gellan gum based microparticles of metoclopromide hydrochloride for intranasal delivery: Development and evaluation. Chem. Pharm. Bull..

[37] Covert R.J., Ott R.D., Ku D.N. (2003). Friction characteristics of a potential articular cartilage biomaterial. Wear.

